# Supplementary material for: Bacterial and Viral Co-Infections in COVID-19 Patients: Etiology and Clinical Impact
Source: Biomedicines. 2024 Sep 27;12(10):2210. doi: 10.3390/biomedicines12102210 (PMC11505336; doi:10.3390/biomedicines12102210)
Supplement: Supplementary file 1 [file biomedicines-12-02210-s001.zip › biomedicines-3183191-supplementary.pdf]

Table S1. Primers and probes used for multiplex PCR for detection of bacterial co-pathogens: sequences, sizes, and concentrations.

Bacteria	Gene	Sequence 5′→3′	PCR Product Size (bp)
Haemophilus influenzae	*hpd*	**F**: GGT TAA ATA TGC CGA TGG TGT TG<br>**R:** TGC ATC TTT ACG CAC GGT GTA	151 bp
Chlamydia pneumoniae	MOMP	**F**: ACA CGA TGC AGA GTG GTT CA<br>**R:** TGT TTA CAG AGA ATT GCG ATA CG	368 bp
Mycoplasma pneumoniae	P1	**F**: ACT CGG AGG ACA ATG GTC AG<br>**R:** CAA ACC CGG TCT TTT CGT TA	483 bp

Table S2. Conventional PCR conditions.

**PCR Conditions**			
**Step**	**Cycles**	**Temperature**	**Time**
1. Initial denaturation	1	94°C	3 min.
2.1 Denaturation<br>2.2 Annealing<br>2.3 Extension	35	(1) 94 °C<br>(2) 61 °C<br>(3) 72 °C	30 sec.<br>30 sec.<br>45 sec.
3. Final extension	1	72 °C	7 min.

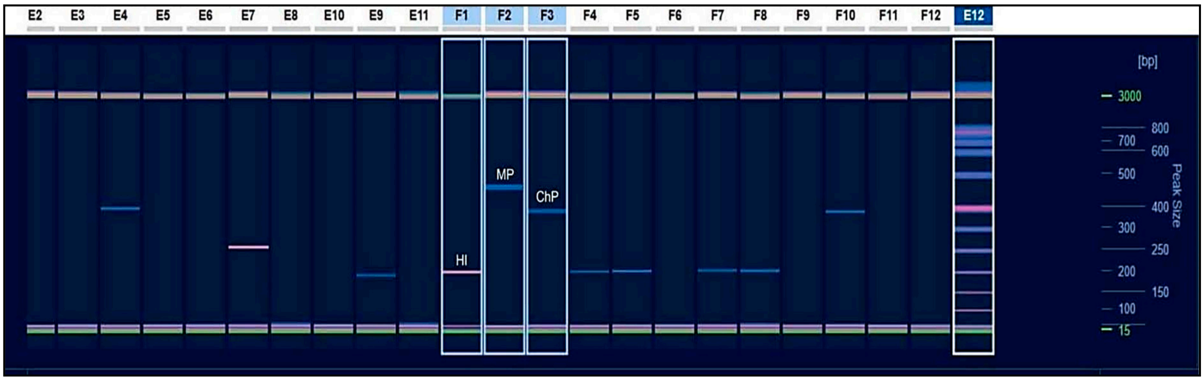

Figure S1. Visualization from automated electrophoretic analysis. (F1—positive control for HI; F2—positive control for MP; F3—positive control for ChPn; E11—negative control (water); E12—molecular marker (50-800 bp); E4, E7, E9, F4, F5, F7, F8, and F10—positive patient samples).

Table S3. Primers and probes used for multiplex real-time PCR to detect respiratory viruses: sequences, sizes, and concentrations.

Virus	Gene	Sequence 5′→3′	Total Concentration	Fluorescent Dye
**Adenovirus (AdV)**	HEXON	**F**: GCC ACG GTG GGG TTT CTA AAC TT<br>**R:** GCC CCA GTG GTC TTA CAT GCA CAT C<br>**P:** TGC ACC AGA CCC GGG CTC AGG TAC TCC GA	500 nM<br>500 nM<br>100 nM	**Texas red**
**Respiratory syncytial virus (RSV)**	M	**F**: GGC AAA TAT GGA AAC ATA CGT GAA<br>**R:** TCT TTT TCT AGG ACA TTG TAY TGA ACA G<br>**P:** CTG TGT ATG TGG AGC CTT CGT GAA GCT	500 nM<br>250 nM<br>50 nM	**HEX**
**Parainfluenza type 1 (PIV1)**	HN	**F**: AGT TGT CAA TGT CTT AAT TCG TAT CAA T<br>**R:** TCG GCA CCT AAG TAA TTT TGA GTT<br>**P:** ATA GGC CAA AGA "T" TGT TGT CGA GAC TAT TCC AA	500 nM<br>500 nM<br>50 nM	**FAM**
**Bocavirus (BoV)**	NS1	**F**: TGC AGA CAA CGC YTA GTT GTT T<br>**R:** CTG TCC CGC CCA AGA TAC A<br>**P:** CCA GGA TTG GGT GGA ACC TGC AAA	500 nM<br>500 nM<br>100 nM	**Cy5**
**Rhinovirus (RV)**	5′NCR	**F**: CPA GCC TGC GTG GC<br>**R:** GAA ACA CGG ACA CCC AAA GTA<br>**P:** TCC TCC GGC CCC TGA ATG YGG C	1000 nM<br>1000 nM<br>100 nM	**HEX**
**Parainfluenza type 2 (PIV2)**	HN	**F**: GCA TTT CCA ATC TAC AGG ACT ATG A<br>**R:** ACC TCC TGG TAT AGC AGT GAC TGA AC<br>**P:** CCA TTT ACC "T"AAG TGA TGG AAT CAA TCG CAA A	750 nM<br>750 nM<br>50 nM	**FAM**
**Human metapneumovirus (HMPV)**	F	**F**: CAA GTG TGA CAT TGC TGA YC<br>**R:** ACT GCC GCA CAA CAT TTA GR<br>**P:** TGG CYG TYA GCT TCA GTC AAT TCA ACA GA	600 nM<br>600 nM<br>100 nM	**HEX**
**Parainfluenza type 1 (PIV3)**	HN	**F**: TGG YTC AAT CTC AAC AAC AAG ATT TAA G<br>**R:** TAC CCG AGA AAT ATT ATT TTG CC<br>**P:** CCC RTC TG"T"TGG ACC AGG GAT ATA CTA CAA A	750 nM<br>500 nM<br>200 nM	**FAM**

Virus	Gene	Sequence 5′→3′	Total Concentration	Fluorescent Dye
**HCoV-229E**	NP	**F**: CAG TCA AAT GGG CTG ATG CA<br>**R:** AAA GGG CTA TAA AGA GAA TAA GGT ATT CT<br>**P:** CCC TGA CGA CCA CGT TGT GGT TCA	750 nM<br>500 nM<br>50 nM	**Texas red**
**HCoV-HKU-1**	NP	**F**: CCT TGC GAA TGA ATG TGC T<br>**R:** TTG CAT CAC CAC TGC TAG TAC CAC<br>**P:** TGT GTG GCG GTT GCT ATT ATG TTA AGC CTG	100 nM<br>750 nM<br>50 nM	**FAM**
**HCoV-NL63**	NP	**F**: GAC CAA AGC ACT GAA TAA CAT TTT CC<br>**R:** ACC TAA TAA GCCTCTTTCTCAACCC<br>**P:** ACC TAA TAA GCC TCT TTC TCA ACC C	250 nM<br>250 nM<br>50 nM	**FAM**
**HCoV-OC43**	NP	**F**: CGA TGA GGC TAT TCC GAC TAG GT<br>**R:** CCT TCC TGA GCC TTC AAT ATA GTA ACC<br>**P:** TCC GCC TGG CAC GGT ACT CCC T	500 nM<br>750 nM<br>50 nM	**HEX**
